# An Unsupervised Text Mining Method for Relation Extraction from Biomedical Literature

**DOI:** 10.1371/journal.pone.0102039

**Published:** 2014-07-18

**Authors:** Changqin Quan, Meng Wang, Fuji Ren

**Affiliations:** 1 AnHui Province Key Laboratory of Affective Computing and Advanced Intelligent Machine, School of Computer and Information, Hefei University of Technology, Hefei, China; 2 Faculty of Engineering, University of Tokushima, 2–1 MinamiJosanjima, Tokushima, Japan; CSIR-Institute of Microbial Technology, India

## Abstract

The wealth of interaction information provided in biomedical articles motivated the implementation of text mining approaches to automatically extract biomedical relations. This paper presents an unsupervised method based on pattern clustering and sentence parsing to deal with biomedical relation extraction. Pattern clustering algorithm is based on Polynomial Kernel method, which identifies interaction words from unlabeled data; these interaction words are then used in relation extraction between entity pairs. Dependency parsing and phrase structure parsing are combined for relation extraction. Based on the semi-supervised KNN algorithm, we extend the proposed unsupervised approach to a semi-supervised approach by combining pattern clustering, dependency parsing and phrase structure parsing rules. We evaluated the approaches on two different tasks: (1) Protein–protein interactions extraction, and (2) Gene–suicide association extraction. The evaluation of task (1) on the benchmark dataset (AImed corpus) showed that our proposed unsupervised approach outperformed three supervised methods. The three supervised methods are rule based, SVM based, and Kernel based separately. The proposed semi-supervised approach is superior to the existing semi-supervised methods. The evaluation on gene–suicide association extraction on a smaller dataset from Genetic Association Database and a larger dataset from publicly available PubMed showed that the proposed unsupervised and semi-supervised methods achieved much higher F-scores than co-occurrence based method.

## Introduction

Because biomedical relations play an important role in biological processes, the study of interactions in the life sciences domain has captured considerable interest. Much effort is currently spent on extracting useful biomedical relationships such as protein–protein interactions or gene–disease associations.

Biomedical relation extraction techniques basically include two branches: interaction database based methods and text mining methods. Interaction database based methods rely on the availability of interaction databases, such as MINT [1], IntAct [2], BIND [3], and SwissProt [4], which predict interactions between entities using sequence, structural, or evolutionary information. Although these databases host a large collection of manually extracted interactions from the literature, manually curated databases require considerable effort and time with the rapid increasing of biomedical literature.

Since most biological facts are available in the free text of biomedical articles, the wealth of interaction information provided in biomedical articles motivated the implementation of text mining approaches to automatically extract biomedical relations. Text mining approaches to relation extraction have shown an evolution from simple systems that rely solely on co-occurrence statistics [5] to complex systems utilizing syntactic analysis or dependency parsing [6]–[8], and machine learning algorithms [9]–[12]. However, most of this research has concentrated on supervised methods requiring large amounts of labeled data. Such annotated resources are expensive to create because the annotation of relations is considerably complicated.

Open Information Extraction started as an effort to approach relation extraction in an unsupervised way by learning regularities and patterns from the web. The Open Information Extraction systems [13]–[15] do not need any manual data or rules, but the relational facts they extract are not disambiguated to entities and relations [16]. As a result, they are hard to be applied in biomedical domain. In addition, Unsupervised Semantic Parsing [17] aims at clustering entity mentions and relation surface forms, and thus generating a semantic representation of the texts on which inference may be used. Some techniques that have been used are Markov Random Fields and Bayesian generative models. These approaches are quite powerful but have very high computational requirements [18].

In this paper, we propose a novel approach for relation extraction. We identify interaction words using polynomial kernel based pattern clustering, which can identify interaction words efficiently in an unsupervised way. The extracted interaction words are combined with phrase structure parsing and dependency parsing for relation extraction, which make full use of both full and partial sentence structure information. Based on the semi-supervised KNN algorithm, we also extend the proposed unsupervised approach to a semi-supervised approach.

In evaluation, we compare the proposed method with several state-of-the-art methods (including supervised and semi-supervised approaches, which used labeled data or manually compiled word list) using a standard biomedical relation corpus.

The experimental results demonstrate the effectiveness of our approach. After that, we employ the proposed approach to predict gene–suicide associations, and show that it achieves much higher F-score than co-occurrence based method.

## Method

### Interaction words identification using pattern clustering

Based on the observation that quite a few biomedical relations can be inferred by interaction words (e.g., IL-6 ***activates*** human gp130; BDNF may ***play a role*** in suicidal behavior), in this section, we present an unsupervised approach for interaction words identification using pattern clustering.

#### Interaction pattern extraction

Windows of limited size around the entities can provide useful clues to identify the roles of the entities within a relation. If two biological entities are co-mentioned in a sentence within certain window, we extract the words between the two biological entities as a candidate pattern. A candidate pattern will be further processed by a filtering process, which filters stopwords, most common words such as “the”, “a”, “that”, and nonenglish words such as numbers or Greek symbols. Any biological entity name contained within a candidate patter would also be filtered out. In addition, patterns with negation words (“no”, “not”, “neither”) are pruned.

#### Interaction words identification

Kernel methods (KMs) are a class of algorithms for pattern analysis, which had been well used in many applications. In this work, we employ KM based interaction pattern clustering for interaction words identification.

KMs approach the problem by mapping the data into a high dimensional feature space. In that space, a variety of methods can be used to find relations in the data [19]. In this work, an interaction pattern polynomial kernel (PK) is generated for pattern clustering. In the basic vector-space model, interaction patterns are represented by a matrix D, whose columns are indexed by the patterns and rows are indexed by the terms.

A pattern 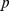 is represented by a row vector, see equation (1). 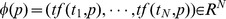
(1)where 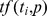 is the frequency of term 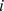 appeared in pattern 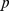.The corresponding kernel is given by the inner product between the feature vectors, see equation (2) and (3).



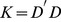
(2)

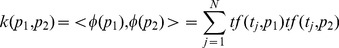
(3)


With the basic kernel, for degree 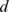 polynomials, the derived polynomial kernel (PK) is defined by equation (4). 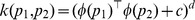
(4)where 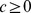 is a constant trading off the influence of higher-order versus lower-order terms in the polynomial. The inner product 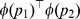 between pattern 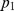 and 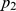 denotes their similarity value.

Based on the definition of polynomial kernel (PK), we can get a PK matrix, and pattern similarities are used to cluster interaction patterns. The pseudo-code of the proposed PK based interaction pattern clustering algorithm is presented in Algorithm 1.

Algorithm 1 first sorts the set of patterns (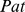) in the descending order of total frequency (Line 1). After sorting, the most common patterns in the corpus appear at the beginning of 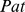, whereas rare instances are shifted to the end. *PoP* function (Line 4) returns and removes the first pattern from 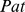. *Assign* function (Line 5) measures the similarity between the vector **p** that corresponds to pattern p and each cluster 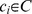. Similarity between **p** and 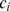 is measured by function 
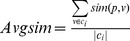
, where 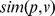 is the similarity between vectors **p** and *v*, 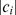 is the number of elements in cluster 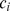. 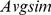 computes the average similarity between **p** and each vector 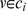 (Line 13).

#### Algorithm 1

PK based interaction pattern clustering algorithm.

**Input**: pattern set (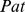), interaction pattern PK matrix (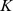), threshold 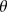


**Output**: pattern clusters 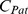


1:   Sort (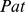)

2:   
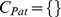


3:   **while** 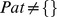 **do**

4:      
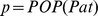


5:      Assign (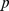, 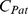, 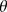)

6:   end while

7:   **return** 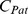


8:   

9:   **function** Assign (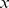, 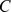, 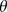)

10:   
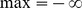


11:   
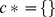


12:   for cluster 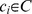 do

13:      
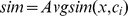


14:      if 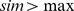


15:          
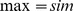


16:          
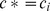


17:      endif

18:   endfor

19:   if 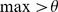 then

20:      
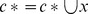


21:   **else**

22:      
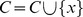


23:   **endif**

If the similarity between **p** and the most similar cluster 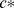 is greater than the threshold 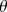, then we merge **p** to 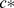. Otherwise, we form a new cluster that contains **p** and append it to 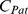. The *while-loop* (Line 3) is repeated until the pattern set (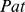) is empty.

Algorithm 1 has a threshold 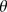, which indirectly specify the number of clusters. We determine 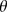 heuristically based on the similarity score distribution of the interaction pattern PK matrix. Specifically, we first define 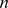 similarity score increment intervals: 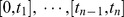 (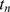 is the max similarity in the PK matrix). We then count the pattern numbers in each interval. When there is a significant drop of pattern number in the current interval (no more than 20 percent of the previous interval), we use that lower limit of the current interval as our threshold 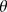.

As most of interaction words are verbs and nouns, we employ the Stanford POS tagging tool (http://nlp.stanford.edu/software/tagger.shtml) to do the POS tagging for patterns and select the verbs which occur more than one times in each cluster. We then normalize the verbs (e.g., activated→activate) and extend interaction words from verbs to nouns (e.g., associate→association) by the SPECIALIST NLP Tools (http://lexsrv3.nlm.nih.gov/Specialist/Home/index.html) to extend the coverage.

### Relation extraction using interaction words and sentence parsing

As stated previously, most of interaction words are verbs and nouns, and because the dependency grammar (DG) views the verb as the structural center of all clause structure, dependency grammar is very fit for relation extraction, and a lot of previous studies extract biomedical relations are based on dependency parsing [7] [20]–[21]. However, dependency parse cannot treat non-local dependencies, and thus rules acquired from the constructions are partial. In addition, one challenge posed by the biological domain is that current systems for parsing do not perform as well on the biomedical narrative as on the newspaper corpora on which they were originally trained [22].

In this work, we combine dependency parsing and phrase structure parsing for relation extraction.

#### Dependency parsing for relation extraction

We assume that if two biological entities are in a relation this should be reflected in their dependencies with the same interaction word. Biomedical dependencies are simply a specific case of dependencies that we would find with a dependency parser.

In the dependency grammar, a syntactic relation between two words 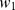 and 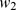 can be described as 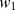 (or 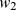) depends on 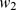 (or 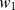). Qiu defined two categories (direct and indirect dependency) to summarize all possible dependencies between two words in sentences [23].

Based on the definition of direct and indirect dependency, we define dependency distance (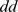) between two words 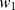 and 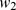 by equation (5).
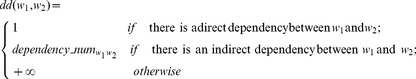
(5)


**Figure 1:**
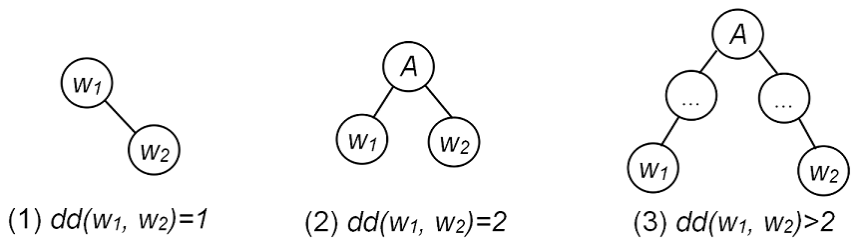
Different dependencies between 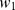 and 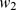.

Equation (5) ignores dependency direction. Both 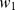 depends on 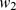 and 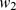
depends on 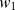 are considered equal. Some examples are given in Figure 1.

Figure 1 (1) illustrates the dependency distance (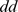) between two words 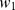 and 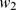 equal to one. Figure 1 (2) shows that both 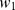 and 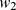 have direct dependencies with word *A*, and the dependency distance (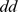) between two words 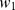 and 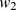 is equal to 2. Figure 1 (3) shows that both 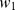 and 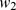 have direct or indirect dependencies with word *A*, and the dependency distance (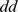) between two words 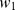 and 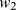 is above 2.

**Figure 2:**
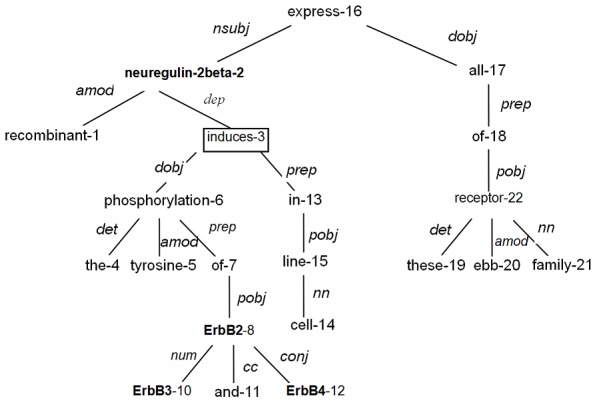
The dependency tree of the sentence ‘Recombinant neuregulin-2beta *induces* the tyrosine phosphorylation of ErbB2, ErbB3 and ErbB4 in cell line express all of these erbb family receptor.’, where words are assigned with word positions (number appended to words), dependency types (italic) appended to edges. Words marked in bold indicate gene/protein names. Words in rectangle indicate interaction words.

Figure 2 shows the dependency tree we obtained for the sentence ‘Recombinant **neuregulin-2beta** induces the tyrosine phosphorylation of **ErbB2**, **ErbB3** and **ErbB4** in cell line express all of these erbb family receptor.’

Based on this dependency tree, we can get the dependency distance (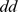) between two words. For instance, there is a direct dependency between ‘**ErbB2**’ and ‘**ErbB3**’, therefore, 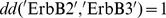; there is an indirect dependency ‘induces—phosphorylation—of—**ErbB2**’ between ‘induces’ and ‘**ErbB2**’, and the dependency number between them is three, therefore 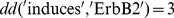.

Given the above description on different dependencies between 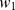 and 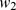, the extraction rules based on dependency parsing is given as follows. (Stanford parser tool is employed for sentence dependency parsing: http://nlp.stanford.edu/software/stanford-dependencies.shtml.)

RD1: Both entity1 and entity2 have direct or indirect dependencies with the same interaction word A, and 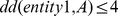, 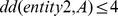.

RD2: If the interaction word is a verb, then this verb should occur between entity1 and entity2 in the sentence.

Rule RD1 extracts paths in the dependency tree that lead from an entity node through an interaction word to another entity node, while limiting the dependency distance between entity node and interaction word node is four or less. This restriction has been found to reduce the number of false paths. Rule RD1 applied on the sentence in Figure 2 extracts the paths as shown in Table 1.

**Table 1:** The extracted paths and relations from the dependency tree (Fig. 2) by Rule RD1.

No.	Path	Relation
1	neuregulin-2beta—*induces*—phosphorylation—of—ErbB2	(neuregulin-2beta, ErbB2)
2	neuregulin-2beta—*induces*—phosphorylation—of—ErbB2—ErbB3	(neuregulin-2beta, ErbB3)
3	neuregulin-2beta—*induces*—phosphorylation—of—ErbB2—ErbB4	(neuregulin-2beta, ErbB4)
4	ErbB3—ErbB2—of —phosphorylation—*induces*—phosphorylation—of—ErbB2	(ErbB3, ErbB2)
5	ErbB4—ErbB2—of —phosphorylation—*induces*—phosphorylation—of—ErbB2	(ErbB4, ErbB2)
6	ErbB3—ErbB2—of —phosphorylation—*induces*—phosphorylation—of—ErbB2—ErbB4	(ErbB3, ErbB4)

*Words marked in bold indicate the focused entities. Italic words indicate interaction words.

As shown from Table 1, there are six relations have been extracted by rule RD1, in which the upper 1–3 are valid, while the lower 4–6 are invalid. Then the restriction of rule RD2 has been found to filter these invalid relations. It reflects that interaction verbs usually occur between two entities they associate (e.g. Protein E1 ***binds*** E2.).

Rule RD2 applied on the sentence in Fig. 2 filters the lower 4–6 invalid relations in Table 1. The upper 1–3 paths are through the interaction word ‘induces’, which indicates their relation type.

For long and complex sentences, the dependency distance between entity node and interaction word node may above four. For instance, in the dependency tree of the sentence ‘A double point mutation in the activation domain of **p53** impaired the ability of this domain to activate transcription and its ability to ***interact*** with both **TAFII40** and **TAFII60**.’, the derived dependency path from ‘p53’ to ‘interact’ is ‘p53—of—domain—in—mutation—impaired—to—transcription—ability—interact’. Because 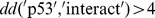, the relations (p53, TAFII40) and (p53, TAFII60) cannot be detected by dependency rules. We apply phrase structure parsing rules to extract the relations that cannot be identified by dependency rules.

### Phrase structure parsing for relation extraction

**Figure 3:**
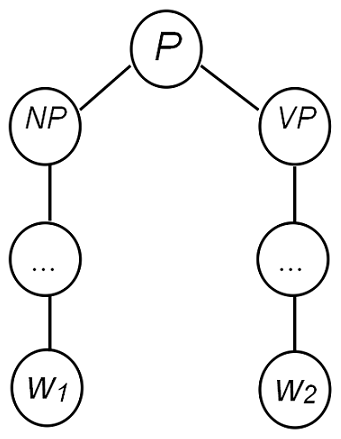
NP+VP phrase structure between 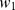 and 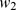.

Phrase structure grammars identify syntactic rather than semantic relations of dependency grammars. Phrase structure parsing is full parsing, which takes into account the full sentence structure. Combined with the interaction characteristics between biological entities, we focus on the type of NP+VP structure, shown by Figure 3.

Figure 3 illustrates that 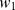 is in an NP structure, 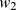 is in a VP structure, and the NP node and VP node have the same parent node. NP+VP structure is able to catch both full and partial sentence structure information. When the NP and VP nodes are the separate direct parents of 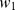 and 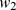, the NP+VP structure represents a partial sentence structure, while when the NP and VP nodes are the separate indirect parents of 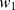 and 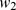, the NP+VP structure represents a wider range structure. When P node is the root node, NP+VP structure represents a full sentence structure.

Because current systems for biomedical narrative parsing are not as reliable as on newspaper corpora, another benefit that we combine dependency parsing and phrase structure parsing is that two different parsers can compensate for each other from the view of system accuracy.

We employ Stanford PCFG phrase structure parsing [24]. The extraction rules based on phrase structure parsing are given as follows.

RP1: Entity1 and entity2 have a NP+VP phrase structure.

RP2: There is an interaction word A in the VP structure of the NP+VP structure in RP1

Rules RP1 and RP2 applied on the sentence ‘A double point mutation in the activation domain of **p53** impaired the ability of this domain to activate transcription and its ability to ***interact*** with both **TAFII40** and **TAFII60**.’ extract the relations (p53, TAFII40) and (p53, TAFII60) that were not be identified by dependency rules.

## Results and Discussion

### Evaluation on protein–protein interactions extraction

We use AImed corpus as the benchmark dataset for protein–protein interactions extraction. AImed corpus is manually developed by Bunescu et al. for protein–protein interaction and protein name recognition [25], which has been used for many protein interaction extraction systems [22] [26]–[30]. AImed corpus consists of 225 Medline abstracts: 200 are known to describe interactions between human proteins, while the other 25 do not refer to any interaction. There are 4084 protein references and around 1000 tagged interactions in this dataset. The corpus and the experimental data can be downloaded from our website (http://a1-www.is.tokushima-u.ac.jp/member/ren/Projects/Unsupervised-biomedical-relation-extraction.htm#userconsent#).

We compare the following four methods on the task of retrieving protein interactions from AImed. The performances are measured using the standard evaluation measures of precision (p), recall (r) and F-score (F), F = 2pr/(p+r). We adopt the evaluation methodology of One Answer per Occurrence in the Document – OAOD (each individual occurrence of a protein interaction has to be extracted from the document) [28].

- **Yakushiji** ***et al.*** **, 2005** [26]: This is a rule based method where linguistic rules were extracted from a relatively small annotated corpus. They demonstrated that their rule extraction method is better than manual-made extraction rules or rules generalized by machine learning techniques.
- **Mitsumori** ***et al.*** **, 2006** [27]: This is a machine learning method. They applied support vector machines (SVMs) to extract protein-protein interaction. An annotated corpus was used for model training.
- **Bunescu** ***et al.*** **, 2006** [22]: This is a kernel based method, which used three types of subsequence patterns to assert relationships between two entities. This is a supervised machine learning method.
- **Giuliano** ***et al.*** **, 2006** [28]: This is a kernel-based machine learning method, which combined global and local context features. This is a supervised machine learning method and solely on shallow linguistic information.
- **Miwa** ***et al.*** **, 2009** [29]: This is a kernel-based machine learning method, which combined several different layers of information from a sentence and its syntactic structures by using several parsers. This is a supervised machine learning method.
- **Erkan** ***et al.*** **, 2007** [30]: This approach is based on the analysis of the paths between two protein names in the dependency parse trees of the sentences. The best performance is achieved by transductive SVM algorithm with edit distance similarity. This is a semi-supervised method.
- **Our proposed I (unsupervised)**: This is a clustering based method, which combines dependency and phrase structure parsing for relation extraction. This is an unsupervised method. In the step of interaction pattern extraction, the window of candidate pattern extraction is set 10 words. The parameters of the polynomial kernel (PK) are 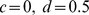 (equation 4). In Algorithm 1, the parameter threshold 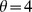 is set by a heuristical method.
- **Our proposed II (semi-supervised)**: This approach combines our proposed I (unsupervised) approach and a semi-supervised KNN (K-Nearest Neighbor) algorithm [30]. In the semi-supervised KNN algorithm, the similarity between two instances is measured by edit-distance that proposed by Erkan et al., 2007 [30]. The semi-supervised KNN algorithm is used for instance classification firstly, and then the interaction words identified by our proposed pattern clustering method and the rules based on dependency parsing (RD1, RD2) and phrase structure parsing (RP1, RP2,) are applied for correcting errors in KNN classification. The parameter of the KNN algorithm is 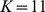. The number of training sentences is 500.

Table 2 shows the results comparison on precision (p), recall (r), and F-score (f) respectively of these approaches.

**Table 2:** Results comparison of protein-protein interactions extraction by different methods.

Method	Descriptions	P	R	F
Yakushiji et al., 2005	Rules based; phrase structure parse; supervised	33.70	33.10	33.40
Mitsumori et al., 2006	SVM based; supervised	54.20	42.60	47.70
Bunescu et al., 2006	Kernel based; svm; supervised	65.00	46.40	54.20
Giuliano et al., 2006	Combination of kernels; global and local context; supervised	60.90	57.20	59.00
Miwa et al., 2009	Combination of kernels; syntactic parseing; svm; supervised	58.70	66.10	61.90
Erkan et al., 2007	Dependency parse; edit distance similarity; transductive SVM; semi-supervised	59.59	60.68	59.96
Our proposed I (Unsupervised)	Clustering based, dependency and phrase structure parse; unsupervised	44.80	71.40	55.10
Our proposed II (Semi-supervised)	Semi-supervised KNN, edit distance similarity, clustering based, dependency and phrase structure parse; semi-supervised	56.60	66.80	60.70

### Evaluation on gene–suicide association extraction

Determining gene-disease associations will enhance the development of new techniques for prevention, diagnosis and treatment of diseases. As the identification of new disease genes based on biomedical experiments require considerable effort and time, increasing attention is being paid to identifying gene–disease associations by mining the amount of biomedical literature.

Suicide receives increasing attention around the world, with many countries developing national strategies for prevention. Hawton and Heeringen analyzed several risk factors for suicide, in which genetic loading is considered one of the most important factors [31]. Costanza *et al*. present the latest neurobiological findings that have been shown to be implicated in suicide completers [32].

In comparison to other diseases, biomedical experiments for finding suicide related genes are much harder to conduct. Many existing databases maintain only a few records on suicide and its related genes. In one of the most well-known gene–disease association databases, Online Mendelian Inheritance in Man (OMIM [33]), suicide has not been recorded and does not have a MIM code. Many other gene-disease databases (DisGeNET [34], KEGG DISEASE [35], and the Human Gene Mutation Database [36]) also return “no results” by querying “suicide”. Consequently, database based methods are hard to be applied for finding gene–suicide relation.

In this section, we report the results applying the proposed method for gene–suicide relation extraction.

#### Corpus

We conduct experiments on two datasets:

**Dataset I**: We used the Genetic Association Database (GAD, http://geneticassociationdb.nih.gov/cgi-bin/index.cgi), which is a curated database of human genetic association studies of complex diseases and disorders. GAD includes summary data extracted from published papers in peer reviewed journals on candidate gene and genome-wide association studies. We search the keyword “suicide” in the search item of “disease” in GAD, and got 199 returned records. We downloaded all of the abstracts of PubMed papers that describe suicide in GAD, and linked each abstract to the suicide related gene that it describes (download date: May 1 2013). This dataset contains 168 PubMed abstracts, and 52 suicide related genes.

**Dataset II**: We downloaded the abstracts from PubMed Central (PMC) Open Access based on the query of “human+suicide” (download date: April 10 2013). This dataset contains 52,126 PubMed abstracts.

We used two databases to get suicide related gene list.

1. GAD gene list. It contains 52 suicide related genes, whose “Assoc?YorN” labels in GAD are null or “Y” in GAD database. The disease phenotype has been annotated by one of phenotypes in the set of {“Suicide”, “Suicide, Attempted”, “bipolar disorder suicide”, “depressed suicide”, “depressive disorder, major sui”, “schizoaffective disorder, alco”, “suicidal ideation”}.
2. GeneCards gene list. It contains 362 suicide related genes, which are obtained by querying “suicide” in GeneCards database (http://www.genecards.org/index.php?path=/Search/keyword/suicide/0/500/score/desc).

#### Text preprocessing

Sentences in abstracts are split by GENIA Sentence Splitter (http://www.genecards.org/index.php?path=/Search/keyword/suicide/0/500/score/desc), which is reported to have an F-score of 99.7 on 200 unseen GENIA abstracts. Gene and protein names are identified by GENIA Tagger (http://www.nactem.ac.uk/GENIA/tagger/) which is reported to have an overall F-score of 71.37% on named entity recognition performance. To normalize the gene names tagged by GENIA Tagger, we use the HUGO Gene Nomenclature Committee (HGNC) database (http://www.genenames.org/cgi-bin/hgnc_stats), which contains 84,584 genes (including gene synonyms). We combined each tagged gene name with its corresponding approved gene symbol.

#### Results

Table 3 shows the experimental results on the two datasets. The baseline method is co-occurrence based method.

**Table 3:** Results comparison with different linguistic parsing and rules.

Suicide related gene list	Method	Dataset I			Datase II		
		P	R	F	P	R	F
GAD gene list	Co-occurrence	54.40	100.00	70.50	14.90	95.10	25.70
	Our proposed (Unsupervised)	71.10	86.50	78.00	25.60	56.10	35.10
	Our proposed (Semi-supervised)	75.35	90.06	82.05	29.55	67.12	41.03
GeneCards	Co-occurrence	54.29	96.50	69.49	24.67	82.03	37.93
	Our proposed (Unsupervised)	63.46	91.67	75.00	28.37	76.62	42.18
	Our proposed (Semi-supervised)	67.80	94.66	79.01	33.28	81.12	47.20

#### Discussion

In this paper we address the problem of biomedical relation extraction based on pattern clustering and sentence parsing. We evaluated our approach on two different tasks. The first task concentrates on protein–protein interactions extraction. Our approach identified interaction words using unsupervised pattern clustering. This is the difference between our approach and the existing methods that used labeled data.

From Table 4, we can see that our proposed unsupervised approach has 21.7%, 7.4% and 0.9% improvement in F-score over Yakushiji *et al.* (2005)'s rule based method, Mitsumori *et al.* (2006)'s SVM based method, and Bunescu *et al.* (2006)'s Kernel based method. All of the three methods are supervised. Our proposed semi-supervised approach has 0.9% improvement in F-score over Erkan et al., 2007's semi-supervised method, and superior to other supervised methods except for Miwa et al., 2009's method.

**Table 4:** The extracted interaction words of protein–protein interaction from Aimed.

Interaction words (from Aimed corpus)
Bind, induce, activate, associate, mediate, block, interact, contain, phosphorylate

In semi-supervised KNN algorithm, each data instance (labeled or unlabeled) is a node that is connected to its *K* nearest neighbor nodes. We experiment different *K* valuses to compare the F-scores with varying sizes of train. The sentences in AIMed dataset ware firstly partitioned into labeled and unlabeled sentence randomly based on the ratio of labeled and unlabeled sentence number (from 1∶5 to 1∶1). The results are the averages over 10 such random runs.

**Figure 4:**
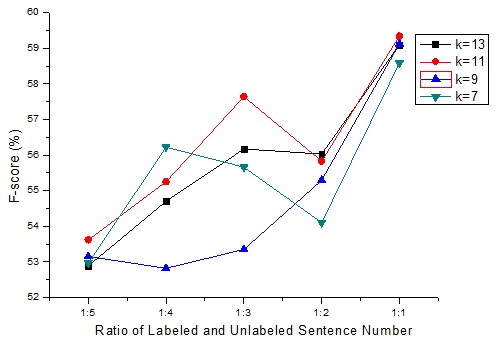
The F-score curves on the AImed corpus with varying sizes of training data with different K values.

Figure 4 shows the F-score curves by using semi-supervised KNN algorithm on the AIMed dataset with varying sizes of training data with different *K* values.

Figure 4 shows that the best F-scores were obtained when 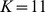 on the average. Based on this result, we compared the semi-supervised KNN algorithm and our proposed semi-supervised approach which combined semi-supervised KNN, pattern clustering, dependency parsing and phrase structure parsing. The parameter of the KNN algorithm is 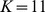.

**Figure 5:**
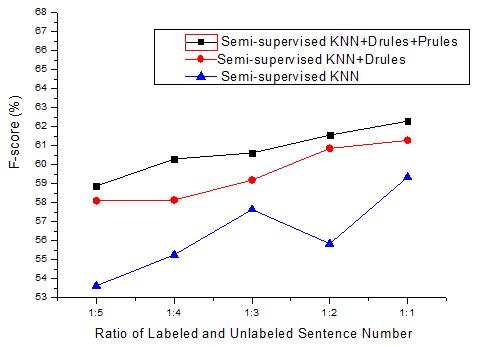
The F-score curves by using semi-supervised KNN algorithm and the proposed semi-supervised approach on the AIMed dataset with varying sizes of training data.

Figure 5 shows the F-score curves by using semi-supervised KNN algorithm and the proposed semi-supervised approach on the AIMed dataset with varying sizes of training data. In the proposed semi-supervised approach, the semi-supervised KNN algorithm was used for instance classification firstly, and then the interaction words identified by our proposed pattern clustering method and the rules based on dependency parsing (RD1, RD2) and phrase structure parsing (RP1, RP2,) were applied for correcting errors in KNN classification.

In Figure 5, ‘Semi-supervised KNN+Drules’ means the approach combined with semi-supervised KNN, pattern clustering, and dependency parsing rules. ‘Semi-supervised KNN+Drules+Prules’ means the approach combined with semi-supervised KNN, pattern clustering, dependency parsing rules, and phrase structure parsing rules. We find that the proposed semi-supervised approach improved the performance of the semi-supervised KNN algorithm greatly. Both dependency parsing rules and phrase structure parsing rules contribute to improving the performance.

Table 4 lists the extracted protein–protein interaction words from AImed dataset by the polynomial kernel (PK) based pattern clustering method, which includes nine interaction verbs.

Based on the identified interaction words, we combined dependency parsing and phrase structure parsing for relation extraction. Table 5 compares the performances of relation extraction with different linguistic rules.

**Table 5:** Results comparison with different linguistic parsing and rules.

Parsing type	Linguistic rules	P	R	F
Dependency parsing	RD1	34.28	75.70	47.19
	RD1+RD2	38.32	72.74	50.20
Phrase structure parsing	RP1+RP2	49.90	39.57	44.14
Dependency parsing + phrase structure parsing	RD1+RD2+RP1+ RP2	44.80	71.40	55.10

From Table 5, we can find that dependency parsing can achieve higher recall, while phrase structure parsing can achieve higher precision. Their combination took the best aspects of each, which achieved higher F-score than either of them. When comparing with previous rule based approaches, the rules defined in our approach are much simpler and easier to implement.

The second task focused on gene–suicide association extraction. Table 6 lists the extracted interaction verbs of gene-suicide relation from Dataset I and Dataset II.

**Table 6:** The extracted interaction verbs of gene-suicide relation from Dataset I and Dataset II.

	Interaction words
Dataset I	Associate, complete, control, increase, consider, homozygote, attempt, suggest, bear, repeat, carry, compare, classify, play, modify, find, indicate, influence, depress, affect, implicate, act, monitor
Dataset II	Attempt, associate, commit, program, increase, compare, complete, message, consider, influence, know, show, use, involve, link, carry, measure, obtain, confer, play, homozygote, implicate, assess, find, function, express, result, combine, contain, act, distinguish, decrease, admit, adopt, report, depress, take, identify, occur, load, risk, indicate, include, reduce, construct, live, confirm, announce, investigate, control, set, prevent, cingulate, liberate, suggest, monitor, hospitalize

From Table 6, we can find that the interaction verbs of gene–suicide relation and of protein–protein interaction are quite different. Based on these interaction verbs, we used the rules based on dependency parsing and phrase structure parsing for gene-suicide relation extraction.As shown in Table 2, when GAD gene list was matched against, our proposed method outperformed co-occurrence based method significantly; the F-scores obtained by the unsupervised method are improved about 7.5% and 9.4% separately on the two datasets; the F-scores obtained by the semi-supervised method are improved about 11.6% and 15. 3% separately on the two datasets; when GeneCards gene list was matched against, our proposed unsupervised method outperformed co-occurrence based method about 5.5% and 4.3% separately on the two datasets; our proposed semi-supervised method outperformed co-occurrence based method about 9.5% and 9.3% separately on the two datasets. However we have to admit that being able to match the list of suicide-related genes present in databases does not equate to finding the appropriate relations within a document, which is one of the limitations of the evaluation approach.

## Conclusions

We have presented a novel approach to extract biomedical relations based on pattern clustering and sentence parsing. Compared to prior work, our approach does not require labeled relation dataset or manually complied word list. The combination of dependency parsing and phrase structure parsing takes the best aspects of each, and achieved higher F-score than either of them. The linguistic rules defined in our approach are quite general and easy to implement in different biomedical relation extraction tasks, including protein-protein interactions, gene-disease association, etc. Based on the semi-supervised KNN algorithm, we extended the proposed unsupervised approach to a semi-supervised approach by combining pattern clustering, dependency parsing and phrase structure parsing rules.

We evaluated our approaches on two tasks. The first is protein- protein interactions extraction. The evaluation on the benchmark dataset (AImed corpus) showed that our proposed unsupervised approach outperformed three supervised methods. The three supervised methods are rule based, SVM based, and Kernel based separately. The proposed semi-supervised approach has 0.9% improvement in F-score over Erkan et al., 2007's semi-supervised method, which obtained the best result on AImed corpus among the existing semi-supervised methods.

The experiments also showed that the combination of dependency parsing and phrase structure parsing took the best aspects of each, and achieved higher F-score than either of them. When comparing with previous rule based approaches, the rules defined in our approach are much simpler and easier to implement.

We also evaluated our approaches on gene–suicide association extraction. They achieved much higher F-score than co-occurrence based method on a smaller dataset from Genetic Association Database (GAD) and a larger dataset from publicly available PubMed.
